# Reference-Adjusted Loss in Life Expectancy for Population-Based Cancer Patient Survival Comparisons—with an Application to Colon Cancer in Sweden

**DOI:** 10.1158/1055-9965.EPI-22-0137

**Published:** 2022-06-14

**Authors:** Therese M.-L. Andersson, Mark J. Rutherford, Bjørn Møller, Paul C. Lambert, Tor Åge Myklebust

**Affiliations:** 1 Department of Medical Epidemiology and Biostatistics, Karolinska Institutet, Stockholm, Sweden.; 2 Biostatistics Research Group, Department of Health Sciences, University of Leicester, Leicester, United Kingdom.; 3 Department of Registration, Cancer Registry of Norway, Oslo, Norway.

## Abstract

**Background:**

The loss in life expectancy, LLE, is defined as the difference in life expectancy between patients with cancer and that of the general population. It is a useful measure for summarizing the impact of a cancer diagnosis on an individual's life expectancy. However, it is less useful for making comparisons of cancer survival across groups or over time, because the LLE is influenced by both mortality due to cancer and other causes and the life expectancy in the general population.

**Methods:**

We present an approach for making LLE estimates comparable across groups and over time by using reference expected mortality rates with flexible parametric relative survival models. The approach is illustrated by estimating temporal trends in LLE of patients with colon cancer in Sweden.

**Results:**

The life expectancy of Swedish patients with colon cancer has improved, but the LLE has not decreased to the same extent because the life expectancy in the general population has also increased. When using a fixed population and other-cause mortality, that is, a reference-adjusted approach, the LLE decreases over time. For example, using 2010 mortality rates as the reference, the LLE for females diagnosed at age 65 decreased from 11.3 if diagnosed in 1976 to 7.2 if diagnosed in 2010, and from 3.9 to 1.9 years for women 85 years old at diagnosis.

**Conclusions:**

The reference-adjusted LLE is useful for making comparisons across calendar time, or groups, because differences in other-cause mortality are removed.

**Impact:**

The reference-adjusted approach enhances the use of LLE as a comparative measure.

## Introduction

The loss in life expectancy, LLE, is a useful measure for summarizing the impact of a cancer diagnosis on an individual's life expectancy (1–8). The measure can also be summarized on a population level, to give an estimate of the population impact of cancer, or the average LLE for a cancer cohort. The LLE is defined as the difference in life expectancy for an individual free of cancer and the life expectancy for a patient with cancer. In practice, the mortality in the general population, usually conditioned on age, year, and sex, is often used for estimating the life expectancy for someone free of cancer as well as for including mortality due to other causes than cancer for the patients with cancer. When interest lies in comparing LLE across population groups, for example, countries, or studying temporal trends over time, any differences in LLE could be due to a combination of differences in cancer mortality and differences in other-cause mortality and general population mortality. It can therefore not be used to draw conclusions regarding differences or changes in cancer prognosis.

When comparisons across groups or over time are of interest, relative survival is usually used (7, 9–13). Relative survival can, under some assumptions, be interpreted as net survival, that is, the survival the patients with cancer would experience if there would be no other possible causes of death. Even though it is useful for comparisons, the interpretation is not very meaningful. Recently, alternative measures were suggested, reference-adjusted crude and all-cause probabilities of death (14, 15). The crude probability of death is the probability of dying due to cancer in the presence of the competing risk of death due to other causes (7, 16, 17). It is therefore easier to interpret as it conveys “real-world” probabilities. However, when comparing crude probabilities between groups, differences could be due to differences in both cancer mortality and other-cause mortality. The reference-adjusted crude or all-cause probability of death both circumvent the limitation of comparability by using a reference mortality for death due to other causes.

In this article, we show how the LLE can be used within the reference-adjusted framework by extending the work of Lambert and colleagues (14). We illustrate the method by investigating temporal trends in LLE of patients with colon cancer in Sweden.

## Materials and Methods

### LLE

The LLE for a patient with cancer is defined as the difference between the life expectancy of an individual with the same characteristics in the general population and the predicted life expectancy of the patient with cancer (1). For an individual with covariate pattern, **X**, this can be expressed as:




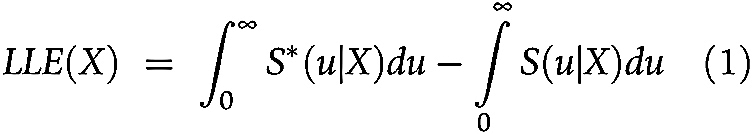




where ${S}^*(t|X)$ is the expected survival for someone with covariate pattern X in the general population and $S(t|X)$ is the survival of a patient with cancer with the same covariate pattern. ${S}^*(t|X)$ is in practice often obtained from population life tables, usually stratified on age, sex, and calendar year. In practice integration is done up to a timepoint, ${t}_{max}$, where both survival functions are assumed to have reached zero. However, the survival curves used for the calculation of LLE are usually not observed until ${t}_{max}$, due to limited follow-up, and need to be extrapolated beyond available data. The expected survival, ${S}^*(t|X)$, can be extrapolated by making assumptions about the future mortality in the general population, often by assuming that the age- and sex-specific expected survival stays the same as in the last available year. The survival of patients with cancer can be extrapolated by expressing the survival as the product between the expected survival and the relative survival, because the relative survival can be more reliably extrapolated than the observed survival (1). Equation (1) is then expressed as:




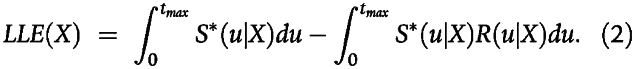




The estimation of LLE with extrapolation of survival using a relative survival approach has previously been described by Andersson and colleagues (1). A flexible parametric relative survival model, using the expected mortality ${h}^*(t|X)$ [the mortality analog of ${S}^*(t|X)$], is used to estimate $R(t|X)$. Flexible parametric relative survival models use splines to model the baseline cumulative excess hazard, and have been extensively described elsewhere (18–20). Equation (2) can be used to obtain estimates of, for example, the LLE for different ages at diagnosis, separately for males and females and over calendar time of diagnosis.

### Marginal estimates

As a summary measure, the marginal LLE can be estimated by averaging the LLE estimates for the cancer cohort:




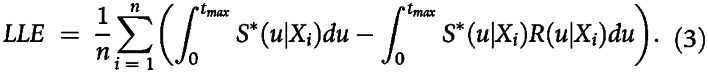




If interest lies in comparisons between groups, the average can be estimated on the basis of the covariate distribution of the other covariates, while keeping the value of the covariate interest fixed. For example, if the interest is to estimate a marginal measure for the hypothetical scenario that all patients had the survival of those diagnosed in year *yy*, the marginal LLE is obtained by:




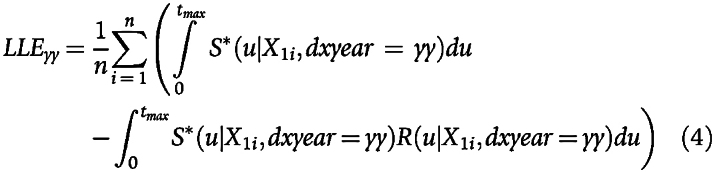




where *X_1_* refers to all covariates except year of diagnosis. This can also be referred to as a standardized summary measure of LLE for those diagnosed in year $yy$, because it is standardized to the covariate distribution of the whole cohort. By estimating this for year $yy$ and another year, say $xx$, the estimates are comparable in terms of age and sex and any other covariates included in *X_1_*. Differences could still, however, be due to differences in expected survival between the different years. Instead of averaging over the full cohort, averaging over a subgroup is also possible, to standardize over the covariate distribution within that subgroup.

### Reference-adjusted LLE

It is often of interest to compare cancer patient survival between groups, for example, countries, regions, or across calendar time. Because the LLE depends on both mortality due to cancer and the expected mortality, there could be differences in LLE between groups even if there are no differences in cancer mortality (i.e., the excess mortality). Lambert and colleagues (14) suggested using a reference population mortality to get a reference-adjusted crude probability of death, where any differences between groups were only due to differences in the cancer mortality (i.e., the excess mortality). This can also be done when estimating LLE. Let us denote the reference population survival by ${S}^{**}(t|X)$, then the reference-adjusted LLE is:




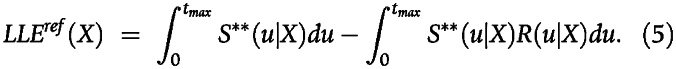




Note that ${h}^*(t|X)$ is still used when fitting the flexible parametric relative survival model used for estimating $R(t|X)$, so that $R(t|X)$ can be interpreted as net survival (21). The estimate obtained from Equation (5) is interpreted as the LLE for an individual with a certain covariate pattern, X, if the mortality due to other causes was the same as that of someone with covariate pattern X in the reference population. It is assumed that the other-cause mortality of patients with cancer is the same as the all-cause mortality in the general population, so the reference-adjusted approach uses the reference rates both for the life expectancy of the general population and in the estimation of life expectancy for the patients with cancer.

When interest is to compare LLE across calendar time, instead of using an external reference population survival, ${S}^{**}(t|X)$, the survival for the general population within a specific year can be used as the reference, ${S}^*(t|{X}_1,year\ = \ yy)$:




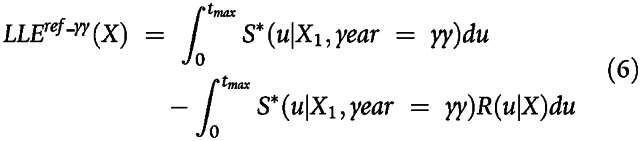




where *X_1_* again refers to all covariates except year of diagnosis. By using the above approach, one can investigate age- and sex-specific temporal trends in LLE, where the differences are only due to differences in the cancer-specific survival. Note that this is not the same ${S}^*(t|X)$ as described in Equation (4), where $dxyear\ = \ yy$ instead of $\ year\ = \ yy$. In the former expression, attained year is used, so the expected rates from multiple years are used even though everyone is assumed to start in a specific year. In the latter approach, rates within a specific year are used throughout the calculation.

### Marginal reference-adjusted LLE

A marginal reference-adjusted LLE can also be obtained in the same way as described previously:




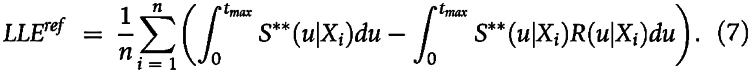




Again, instead of using an external reference population survival, the survival from a single year can be used,




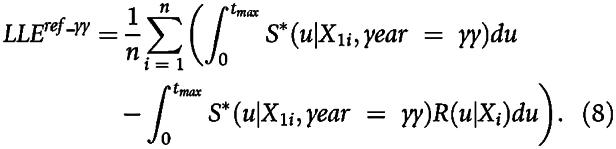




This will give an average LLE in the cohort (given the observed covariate distribution), if the expected survival across calendar year is the same as the general population survival in year $\ yy$.

When interest lies in comparing across calendar years, for example, comparing year *xx*1 and *xx*2, instead of an overall average, the reference population survival can be obtained from a specific year, for example, *yy*, while the relative survival for years xx1 and xx2 can be used. For instance, say we wish to compare calendar years 1980 and 1990 while using the year 2000 for the reference rates, then we would use the relative survival for years 1980 and 1990, respectively, but use the year 2000 for the reference rates. To make sure that any differences are not due to differences in covariate distribution, the measures can be standardized as before, and the formula below can be used,




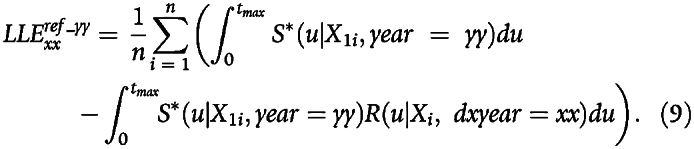




By varying the year *xx* in Equation (9), as described above, but keeping the same year for the reference population survival (i.e., year *yy*), the reference-adjusted and standardized LLE estimates are comparable both in terms of the covariate distribution (such as age and sex) and the expected survival. Instead of standardizing over the covariate distribution in the full cohort, it is also possible to standardize to the distribution in a subgroup, for example the age and sex distribution of the most recent year by averaging over the patients diagnosed in the most recent year only.

### Material

All diagnoses of colon cancer in the Swedish Cancer Register were identified. In this study, we included patients with colon cancer diagnosed at ages 50 to 99 during the years 1976 to 2010. Cancer cases diagnosed incidentally at autopsy were excluded, and only the first recorded colon cancer diagnosis per individual was considered, and the final cohort consisted of 99,227 patients with colon cancer. The individuals were followed from the date of diagnosis until death, and censored at the end of follow-up which was December 31, 2017. Mortality rates in the Swedish general population were obtained from the Human Mortality Database, stratified by age, year, and sex, and available up until year 2018. This study was approved by the Regional Ethical Review Board in Stockholm (2017/641-31/1, 2019-01913, 2020-06544). Informed consent from study subjects was not required for the current study. The study was conducted in accordance with the Declaration of Helsinki.

### Statistical analysis

A flexible parametric relative survival model was fitted, including the covariates age at diagnosis, calendar year of diagnosis, and sex. Age and year were included as continuous functions using restricted cubic splines with 4 degrees of freedom. The baseline log cumulative excess hazard was modeled with 5 degrees of freedom, and time-varying effects (nonproportional hazards) were included for all covariates with 3 degrees of freedom for each term. Two-way interactions were included between age, sex, and year, but using splines with 2 degrees of freedom for age and year.

On the basis of the model, the life expectancy among patients with cancer, life expectancy in the general population, and the LLE was estimated for each age at diagnosis, year of diagnosis, and sex, that is, estimates of LLE conditional on covariates. These conditional estimates were estimated in three ways, (i) using the same expected mortality rates as used when modeling, (ii) reference-adjusted life expectancies and LLE using the age- and sex-specific mortality rates from the general population in 1976, and (iii) reference-adjusted life expectancies and LLE using the age- and sex-specific mortality rates from the general population in 2010. In most situations, (iii) would be more sensible than (ii), but we use (ii) to illustrate the impact of using different reference mortality rates.

Marginal, or standardized, life expectancies and LLE were also estimated, using different approaches. First, the overall marginal LLE in the cancer cohort, as shown in Equation (3). Second, using the age and sex distribution in the whole cohort, but changing the expected survival and the relative survival to be the survival in 1976 and in 2010 [as in Equation (4)]. Marginal reference-adjusted LLE was also estimated using 1976 and 2010 mortality rates [as in Equation (8)], and finally using the general population mortality rates from 1976 and the relative survival of 2010, and vice versa [as described in Equation (9)].

### Data availability

These data were retrieved from the Swedish Cancer Registry, at the National Board of Health and Welfare (NBHW). The data used for this study may not, according to the agreement with NBHW, be shared by the authors to a third party. It is accessible by application to the NBHW.

## Results

**Figure 1.**
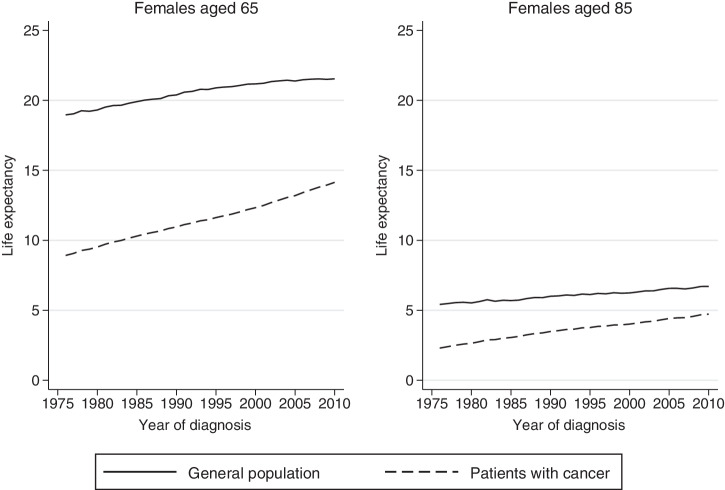
Life expectancy of female colon cancer patients in Sweden diagnosed during the years 1976 to 2010 at ages 65 and 85, and the corresponding life expectancy in the general population.

**Figure 2.**
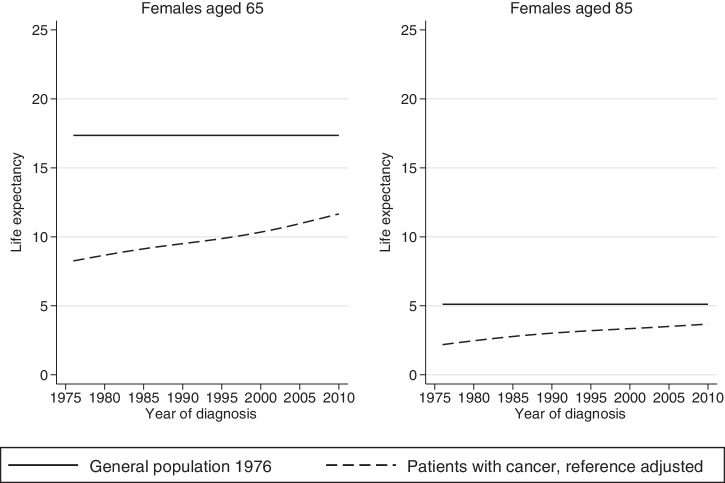
Reference-adjusted life expectancy of female colon cancer patients in Sweden diagnosed during the years 1976 to 2010 at ages 65 and 85, and the corresponding life expectancy in the general population, using the expected mortality rates from year 1976.

**Figure 3.**
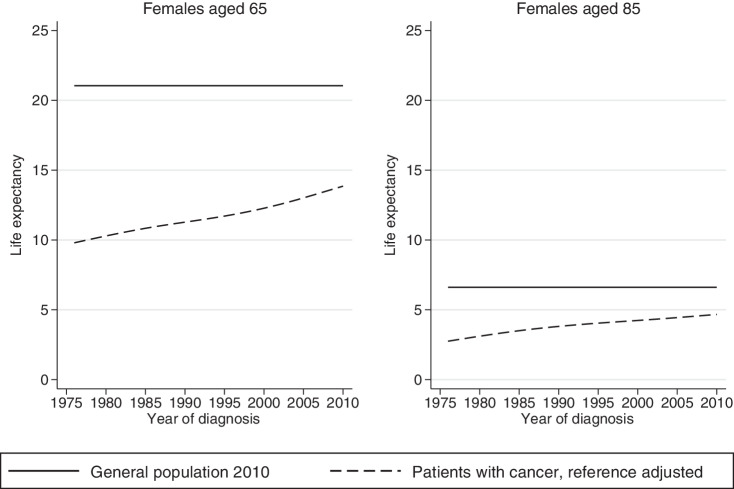
Reference-adjusted life expectancy of female colon cancer patients in Sweden diagnosed during the years 1976 to 2010 at ages 65 and 85, and the corresponding life expectancy in the general population, using the expected mortality rates from year 2010.

Figure 1 shows the life expectancy for patients with colon cancer over calendar year of diagnosis, for females and ages 65 and 85 at diagnosis. The life expectancy for females in the general population, for the same calendar years and ages are also shown. The life expectancy of patients with colon cancer had improved over time, from 8.9 years in 1976 to 14.1 in 2010 for females ages 65 at diagnosis, and 2.3 to 4.7 for females ages 85 at diagnosis. The life expectancy in the general population has also improved, from 19.0 to 21.5 years for females ages 65 and from 5.4 to 6.7 for females ages 85. It is therefore difficult to know how much the improvement in life expectancy for patients with cancer is due to improvement in cancer survival and how much is due to a general improvement in survival due to other causes of death. If instead a reference-adjusted approach with the 1976 mortality rates is used, it is assumed that there are no improvements in life expectancy in the general population, and the general population life expectancy is 17.4 and 5.1 for a 65-year-old and 85-year-old woman, respectively (Fig. 2). The reason why these numbers are not the same as for year 1976 in the standard approach (Fig. 1), is that the standard approach uses attained calendar year. So, the life expectancy for a woman in the general population ages 65 in 1976 would be based on the survival probability for 65-year-old woman in 1976, then the survival probability of a 66 years old in year 1977, then a 67 year old in 1978, etc. For a patient with colon cancer, the life expectancy, using the reference-adjusted approach with 1976 mortality rates, increases from 8.3 to 11.7 for a female 65 years old at diagnosis, and from 2.2 to 3.7 for females ages 85 at diagnosis. These estimates can be interpreted as the life expectancy the patients with cancer would have had if the mortality due to other causes (measured using the general population mortality) would not have changed since 1976. The increase is explained by changes in cancer survival, and not influenced by trends in the general population mortality. If instead the mortality rates from year 2010 are used, the general population life expectancy is 21.0 and 6.6 for a 65-year-old and 85-year-old woman, respectively (Fig. 3). The life expectancy for female patients with colon cancer increases from 9.8 to 13.9 for those 65 years at diagnosis and 2.7 to 4.7 for age 85, from year 1976 to 2010.

**Figure 4.**
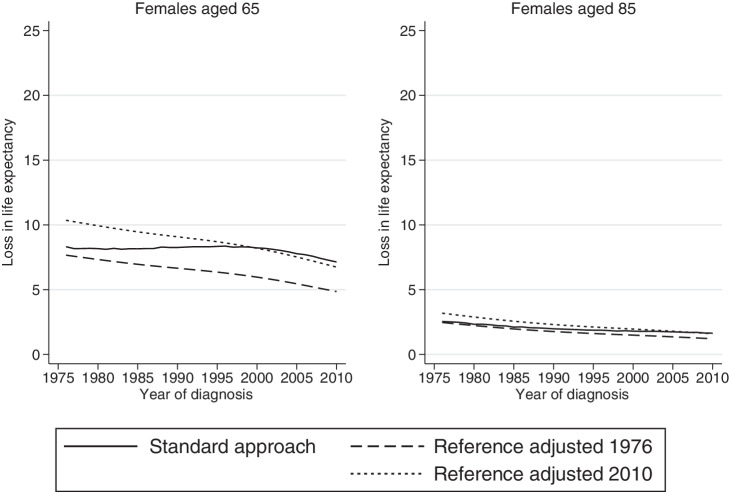
LLE for patients with colon cancer in Sweden diagnosed during the years 1976 to 2010 at ages 65 and 85, using the standard approach and a reference-adjusted approach with two different years as reference (1976 and 2010).

The LLE, that is, the difference in life expectancy in general population and the life expectancy for patients with cancer, is shown in Fig. 4. The LLE based on the life expectancies shown in Fig. 1 is fairly constant over time, even though the life expectancy of patients with cancer is increasing the LLE does not decrease markedly because the life expectancy in the general population is also increasing. In 1976, the LLE is 10.0 for a female patient with colon cancer ages 65 at diagnosis and 3.1 for age 85, this is 7.4 and 2.0, respectively in year 2010. The reference-adjusted LLE on the other hand is decreasing, both when 1976 rates and 2010 rates are used as reference rates, although the values are different. Because these estimates are not influenced by changes in the general population mortality, they can be used to make fair comparisons across time. If the mortality rates due to other causes would have stayed constant at the levels observed in 1976, but the observed improvement in colon cancer survival would persist the LLE would decrease from 9.1 to 5.7 years for 65-year-old females, and from 2.9 to 1.4 years for women 85 years old at diagnosis. If rates from 2010 are used, these numbers are 11.3 to 7.2 for those ages 65 at diagnosis and 3.9 to 1.9 for those ages 85.

The marginal LLE for the entire cohort was 5.8 years (Table 1), with life expectancy of 7.7 years compared with 13.5 in the general population. Using a reference-adjusted approach with 1976 expected rates as reference these numbers are 4.7, 6.5, and 11.2, respectively, and with 2010 expected rates 6.1, 8.0, and 14.1. Reference-adjusted measures also allows comparisons across time, that are not influenced by differences in background mortality and changes in age and sex distribution. The reference-adjusted marginal LLE, using 1976 expected rates, is 6.0 years if the patients experience the excess mortality of those diagnosed in 1976 and 3.5 years if experiencing the excess mortality of those diagnosed in 2010. These numbers are comparable in the sense that they are not influenced by any changes in mortality due to other causes, changes in life expectancy of the general population or by changes in the distribution of age and sex of the patients with cancer. Using expected rates from 2010, the marginal reference-adjusted LLE is 7.7 years with excess mortality of 1976 and 4.7 years with excess mortality of 2010.

**Table 1.** Marginal life expectancy (LE) and loss in life expectancy (LLE) for patients aged 50 to 99 with colon cancer in Sweden during the years 1976 to 2010, using different reference-adjusted approaches and different years as reference.

Year used for reference adjustment	Year used for excess mortality rates	LE without cancer	LE of cancer patients	LLE	Corresponding equation
None	All	13.5	7.7	5.8	3
1976	All	11.2	6.5	4.7	8
1976	1976	11.2	5.2	6.0	9
1976	2010	11.2	7.6	3.6	9
2010	All	14.1	8.0	6.1	8
2010	1976	14.1	6.4	7.7	9
2010	2010	14.1	9.4	4.7	9

## Discussion

The life expectancy and LLE of patients with cancer is a useful measure for summarizing cancer patient survival (1–8). It is easy to interpret, and can be presented on an individual or population level. However, it is less useful for making comparisons across groups or over time, because the life expectancy and the LLE depend on both the mortality due to cancer and the expected mortality. It is therefore difficult to know how much of observed differences in LLE are due to differences in other-cause mortality and how much is due to differences in cancer mortality. We have demonstrated an alternative measure, the reference-adjusted LLE, where differences in other-cause mortality are removed by using a reference mortality rate. This builds on work by Lambert and colleagues (14) that demonstrated the reference-adjusted approach for estimation of crude probabilities of death and all-cause death of patients with cancer within a relative survival setting.

In this article, we investigated how the life expectancy and LLE of patients with colon cancer have changed over calendar time in Sweden. The life expectancy for a woman diagnosed with colon cancer at age 65 has changed from 8.9 to 14.1 between 1976 and 2010, and this increase is due to both improvement in the mortality due to cancer and a general decrease in mortality in the Swedish population. If there would have been no change in the general population mortality since 1976, the life expectancy would have increased from 8.3 to 11.7 between 1976 and 2010, solely due to improvements in colon cancer mortality. Even though this is a hypothetical scenario, it can be used to isolate the impact of the improvements in cancer mortality on the life expectancy and LLE of patients with cancer, and enable fair comparisons across time. Improvements in survival of patients with colon cancer have been observed in many studies, and suggested to be due to improvement in patient management, adjuvant treatments, surgical techniques, and availability of endoscopic investigation (2, 10, 22–26).

The approach demonstrated in this article, can also be used for other type of comparisons, for instance across subgroups or countries. For making comparisons across calendar time or across groups, it is also important to take into account that the age distribution might differ. This is also true for other covariates, such as sex, however often of less importance than age. We have shown sex- and age-specific estimates across time, which circumvents this problem. However, a single summary measure is often also of interest, and can be obtained using marginal estimates. By standardizing to the age and sex distribution in the full cohort, or a particular subgroup (or year) of interest, summary measures can be obtained for different years.

We chose to use two different reference expected rates, the rates in the Swedish general population in year 1976 and 2010. Because of great improvement in general population mortality in Sweden between 1976 and 2010, the rates are fairly different. The results from the two different reference expected rates clearly show how much the estimated LLE can be influenced by the choice of reference. The magnitude of the influence depends on how different the reference rates are, similarly how different the reference-adjusted LLE is from the “true” LLE depends on how different the reference rates and the population mortality rates are. The reference-adjusted LLE has to be interpreted with this in mind, and the main purpose is for making comparisons. However, this is not different to other type of standardization used to enable comparability; for example, age standardization will also give different estimates depending on which age standardization is used.

The best choice of reference rates depend on the research question. In general, we think it will be more sensible to use reference rates from a recent year when the aim is to make comparisons across time. When interest lies in comparisons across groups or countries, it could be of interest to use the expected rates from one group or country of particular interest. This would give the “true” observed LLE for that group, and the reference adjusted of the other group (so hypothetical LLE) if it had the expected rates of the first group. However, if there were no specific group that is of more interest then another alternative would be to use an average expected rate, averaged over the groups that are being compared. No recognized international reference rates, like the International Cancer Survival Standard (ICSS) weights for age standardization, currently exist, but such standard would be useful for future international benchmarking studies.

In conclusion, LLE is a useful summary measure of cancer patient survival, but not ideal for making comparisons across groups, because differences are not only due to differences in cancer mortality. The reference-adjusted LLE can be presented in addition to the standard LLE. It is a useful alternative for making comparisons across calendar time, or groups, because any differences in other-cause mortality are removed.
